# Systemic Microvascular Dysfunction and Inflammation after Pulmonary Particulate Matter Exposure

**DOI:** 10.1289/ehp.8413

**Published:** 2005-10-13

**Authors:** Timothy R. Nurkiewicz, Dale W. Porter, Mark Barger, Lyndell Millecchia, K. Murali K. Rao, Paul J. Marvar, Ann F. Hubbs, Vincent Castranova, Matthew A. Boegehold

**Affiliations:** 1 Department of Physiology and Pharmacology, and; 2 Center for Interdisciplinary Research in Cardiovascular Sciences, West Virginia University School of Medicine, Morgantown, West Virginia, USA; 3 Pathology and Physiology Research Branch, Health Effects Laboratory Division, National Institute for Occupational Safety and Health, Morgantown, West Virginia, USA

**Keywords:** arteriole, endothelium, microcirculation, myeloperoxidase, oxidative stress, particulate matter, polymorphonuclear leukocyte, systemic, venule

## Abstract

The epidemiologic association between pulmonary exposure to ambient particulate matter (PM) and cardiovascular dysfunction is well known, but the systemic mechanisms that drive this effect remain unclear. We have previously shown that acute pulmonary exposure to PM impairs or abolishes endothelium-dependent arteriolar dilation in the rat spinotrapezius muscle. The purpose of this study was to further characterize the effect of pulmonary PM exposure on systemic microvascular function and to identify local inflammatory events that may contribute to these effects. Rats were intratracheally instilled with residual oil fly ash (ROFA) or titanium dioxide at 0.1 or 0.25 mg/rat 24 hr before measurement of pulmonary and systemic microvascular responses. *In vivo* microscopy of the spinotrapezius muscle was used to study systemic arteriolar responses to intraluminal infusion of the Ca^2+^ ionophore A23187 or iontophoretic abluminal application of the adrenergic agonist phenylephrine (PHE). Leukocyte rolling and adhesion were quantified in venules paired with the studied arterioles. Histologic techniques were used to assess pulmonary inflammation, characterize the adherence of leukocytes to systemic venules, verify the presence of myeloperoxidase (MPO) in the systemic microvascular wall, and quantify systemic microvascular oxidative stress. In the lungs of rats exposed to ROFA or TiO_2_, changes in some bronchoalveolar lavage markers of inflammation were noted, but an indication of cellular damage was not found. In rats exposed to 0.1 mg ROFA, focal alveolitis was evident, particularly at sites of particle deposition. Exposure to either ROFA or TiO_2_ caused a dose-dependent impairment of endothelium-dependent arteriolar dilation. However, exposure to these particles did not affect microvascular constriction in response to PHE. ROFA and TiO_2_ exposure significantly increased leukocyte rolling and adhesion in paired venules, and these cells were positively identified as polymorphonuclear leukocytes (PMNLs). In ROFA- and TiO_2_-exposed rats, MPO was found in PMNLs adhering to the systemic microvascular wall. Evidence suggests that some of this MPO had been deposited in the microvascular wall. There was also evidence for oxidative stress in the microvascular wall. These results indicate that after PM exposure, the impairment of endothelium-dependent dilation in the systemic microcirculation coincides with PMNL adhesion, MPO deposition, and local oxidative stress. Collectively, these microvascular observations are consistent with events that contribute to the disruption of the control of peripheral resistance and/or cardiac dysfunction associated with PM exposure.

Epidemiologic studies have suggested that exposure to particulate air pollution may result in as many as 100,000 premature deaths per year in the United States (Schwartz et al. 2002). Multiple studies over a broad range of geographical locations indicate that for each 10 μg/m^3^ increase in ambient particulate matter (PM), the daily mortality rate is augmented by approximately 1–5% (Pope et al. 2002; Schwartz et al. 1996). Mortality due to cardiovascular complications after acute PM exposure comprises a significant component of all-cause mortality (Goldberg et al. 2001; Samet et al. 2000). The U.S. population continues to grow; at least two-thirds of our population is obese or overweight (Hedley et al. 2004), and now one-third is hypertensive (Fields et al. 2004). Children and senior citizens comprise approximately 40% of the total U.S. population, and this figure is projected to swiftly increase in the coming years (U.S. Census Bureau 2004.). A major concern is that despite the ongoing growth of the most susceptible populations, the mechanisms by which PM increases morbidity and mortality remain largely unknown.

Three prominent hypotheses have been advanced to explain how pulmonary PM exposure can elicit a cardiovascular response (Brook et al. 2004; Nemmar et al. 2002; Oberdorster et al. 2004). The first hypothesis proposes that PM deposited in the lung acts through a neural mechanism to alter central nervous system function. In the lung, nociceptive neurons are stimulated by residual oil fly ash (ROFA) (Veronesi et al. 2000). Cardiac autonomic function is also altered by PM exposure, suggesting that central input to the heart is altered (Chen and Hwang 2005). Acute electrocardiographic changes after PM exposure are also suggestive of an activated neural mechanism (Wichers et al. 2004). The second hypothesis proposes that PM deposited in the lung gains access to the systemic circulation and directly interacts with target tissues. After exposure, PM deposition has been reported in a variety of extrapulmonary tissues, including the blood, ventricular microvascular walls, liver, spleen, heart, and brain (Calderon-Garciduenas et al. 2001; Kreyling et al. 2002; Nemmar et al. 2001, 2002; Oberdorster et al. 2002, 2004). No study has shown *in vivo* that the presence of particles within a peripheral tissue is detrimental to its function, but several lines of evidence support this hypothesis. In Mexico City canines, PM deposition was found in the cardiac arteriolar wall where polymorphonuclear leukocyte (PMNL) margination and micro-thrombi were also observed (Calderon-Garciduenas et al. 2001). Although not truly representative of PM exposure and subsequent deposition, treatment of cells or tissues *in vitro* with PM causes cytokine and tumor necrosis factor-alpha (TNF-α) production, cytotoxicity via endotoxins, oxidative stress, and smooth muscle relaxation (Bagate et al. 2004; Dye et al. 1997; Li et al. 2003; Osornio-Vargas et al. 2003; van Eeden et al. 2001). The third hypothesis proposes that PM deposited in the lung initiates a local inflammatory response that develops into a systemic inflammatory response, characterized by alterations in circulating factors and cells associated with inflammation. Pulmonary inflammation after PM exposure is well documented by our laboratory and many others, in animals as well as humans (Dreher et al. 1997; Nurkiewicz et al. 2004; Schaumann et al. 2004). Circulating interleukin (IL)-1 and IL-6 are elevated in humans exposed to PM (van Eeden et al. 2001). IL-1, TNF-α, and the immune-related transcription factor nuclear factor κB are elevated in the brain tissue of mice exposed to PM (Campbell et al. 2005). Furthermore, blood samples from healthy humans exposed to PM reveal elevations in immature PMNL, neutrophils, and platelets (Salvi et al. 1999; Tan et al. 2000).

Although the evidence for these three hypotheses is substantial, and end points have been identified in some cases, the ultimate basic mechanisms responsible for perturbations in a given system are unclear. We have previously shown that PM exposure impairs or abolishes systemic endothelium-dependent arteriolar dilation and dramatically increases venular leukocyte adhesion and rolling (Nurkiewicz et al. 2004). As part of a logical and methodic progression toward identifying these basic mechanisms, we undertook the present study to expand our previous findings in the systemic microvasculature and better characterize the remote effects of pulmonary PM exposure on the spinotrapezius muscle microcirculation. We hypothesized that after PM exposure, events linked to inflammation, such as hemoprotein deposition and oxidative stress, should be present at the microvascular level.

## Experimental objectives.

Our first objective was to determine if alteration of systemic microvascular function can occur after pulmonary PM exposure at levels that fail to cause gross pulmonary toxicity. Rats were exposed by intratracheal (IT) instillation to various doses of ROFA, titanium dioxide, or saline. We assessed pulmonary inflammation and damage by measuring bronchoalveolar lavage (BAL) parameters and evaluated systemic microvascular function by intravital microscopy of spinotrapezius muscle arterioles. Microvascular reactivity was determined by measurement of dilator responsiveness to endothelial stimulation.

Our second objective was to determine if the effects of pulmonary PM exposure on systemic microvascular reactivity to vasodilators are due to an enhanced vasopressor effect via modification of adrenergic responsiveness. Rats were treated with saline or 0.25 mg ROFA by IT instillation. Spinotrapezius muscle arterioles were studied by intravital microscopy. Adrenergic responsiveness was determined by measurement of constrictor responsiveness after α_1_-adrenergic receptor stimulation.

Our third objective was to identify events in the lung, blood, and systemic microcirculation that are consistent with inflammation after PM exposure. Rats were treated with saline or various doses of ROFA or TiO_2_ by IT instillation. We evaluated the number of rolling and adherent leukocytes in spinotrapezius muscle venules, lung histology, muscle histology, microvascular mRNA levels, myeloperoxidase (MPO) deposition, and oxidative stress in the spinotrapezius muscle microvascular wall.

## Materials and Methods

### PM preparation.

ROFA was collected from a precipitator at Boston Edison Co., Mystic Power Plant number 4 (Everett, MA). ROFA particle size and elemental composition from this source have been previously characterized (Antonini et al. 2002; Roberts et al. 2004). ROFA particles were of respirable size with a count mean diameter of 2.2 μm. We used TiO_2_ (mean diameter of 1 μm; Aldrich, Milwaukee, WI) to determine if any ROFA effects were substance specific. ROFA and TiO_2_ samples (suspended in 300 μL sterile saline) were sonicated for 1 min before IT instillation.

### Experimental animals.

Male Sprague Dawley rats (7–8 weeks of age) were purchased from Harlan Sprague Dawley (Indianapolis, IN) and housed at the West Virginia University Health Sciences Center in an animal facility approved by the Association for Assessment and Accreditation of Laboratory Animal Care. To ensure that all methods were performed humanely and with regard for alleviation of suffering, all experimental procedures were approved by the West Virginia University Animal Care and Use Committee.

### IT instillation.

Rats were lightly anesthetized by an intraperitoneal (ip) injection of sodium methohexitol and IT instilled with ROFA (0.1 or 0.25 mg/rat) according to previously established methods (Brain et al. 1976). We have previously shown that these doses partially impair or completely abolish endothelium-dependent arteriolar dilation in the rat spinotrapezius muscle (Nurkiewicz et al. 2004). Rats in the vehicle control group were IT dosed with 300 μL sterile saline. Rats in the particle control group were dosed with TiO_2_ (0.1 or 0.25 mg/rat). After IT instillation, all rats recovered for 24 hr before BAL, histology, or intravital microscopy experiments.

### Collection of BAL samples for measurement of pulmonary inflammation and damage.

Rats were euthanized with sodium pentobarbital (≥ 100 mg/kg, ip). A tracheal cannula was inserted, and BAL was performed through the cannula using ice-cold Ca^2+^/Mg^2+^-free phosphate-buffered saline as previously described (Nurkiewicz et al. 2004).

### BAL fluid lactate dehydrogenase (LDH) activity and albumin protein assays.

BAL fluid LDH activities were determined as a marker of cytotoxicity, and albumin concentrations were determined as an indicator of the integrity of the alveolar air–blood barrier. Both assays were measured as previously described (Nurkiewicz et al. 2004).

### Alveolar macrophage (AM) chemiluminescence (CL).

AM CL was determined as previously described (Nurkiewicz et al. 2004) to evaluate reactive oxygen species production by AM.

### Histology and immunohistochemistry.

To more thoroughly identify pulmonary inflammation, microscopic sections of lungs from rats treated with saline or exposed to 0.1 mg ROFA were evaluated. Lung tissue sections were evaluated by a board-certified veterinary pathologist for morphologic alterations. Semi-quantitative pathology scores were calculated for alveolar inflammation in each slide. The pathology score was the sum of numeric conversion of the severity (none, minimal, mild, moderate, marked, or severe) and distribution (none, focal, locally extensive, multifocal, multifocal and coalescent, or severe) of tissue alterations to produce a pathology score on a scale of 0–10 (Porter et al. 2001).

To characterize cell types associated with systemic microvascular inflammation, histologic analysis was performed on the spinotrapezius muscle of rats 24 hr after exposure to ROFA or TiO_2_ (0.1 or 0.25 mg for each particle type). The muscle was removed from the rat, fixed immediately in 10% formalin, processed, and embedded in paraffin. Paraffin sections stained with hematoxylin and eosin (H&E) allowed positive identification of neutrophils and eosinophils.

To localize the hemoprotein MPO in the spinotrapezius muscle of rats 24 hr after treatment with saline or 0.25 mg ROFA, immunohistochemistry was performed as previously described (Eiserich et al. 2002). Sections were deparaffinized and, after microwave antigen retrieval in citrate buffer pH 6, were incubated overnight at 4°C with a polyclonal antibody against MPO (1:500; Calbiochem, EMD Biosciences Inc., La Jolla, CA). An Alexa 488 fluorescent-conjugated goat anti-rabbit secondary antibody (Molecular Probes, Eugene, OR) was used to localize MPO. After counter-staining the nuclei with diamidinophenylin-dole (DAPI; Molecular Probes), sections were examined with a Zeiss LSM 510 laser scanning confocal microscope system (Carl Zeiss Inc., Thornwood, NY).

### Reverse transcriptase–polymerase chain reaction (RT-PCR).

The left and right spinotrapezius muscles were excised from rats 24 hr after treatment with saline or 0.25 mg ROFA, and the microcirculation was dissected from the surrounding skeletal muscle. The pooled microvascular samples from an individual rat were stored in RNAlater (Ambion, Austin, TX) at 4°C for total RNA isolation. Total RNA was isolated using an Array Pure Nano-scale RNA Purification Kit (Epicentre, Madison, WI) and analyzed as previously described (Rao et al. 2004).

### Intravital microscopy.

Rats were anesthetized with sodium thiopental (100 mg/kg, ip) and placed on a heating pad to maintain a 37°C rectal temperature. The trachea was intubated to ensure a patent airway, and the right carotid artery was cannulated to measure arterial pressure. The right spinotrapezius muscle was then exteriorized, superfused with an electrolyte solution, and prepared for microscopic observation as previously described (Nurkiewicz et al. 2004).

The animal preparation was then transferred to the stage of an intravital microscope. Video images were displayed and videotaped for off-line analysis. During videotape replay, arteriolar inner diameters were measured and venular leukocyte adhesion was quantified.

### Experimental protocols.

#### Protocol 1.

Arteriolar endothelium-dependent dilation was evaluated by assessing the capacity for Ca^2+^-dependent endothelial nitric oxide formation in response to intraluminal infusion of the calcium ionophore A23187 (Sigma Chemical Co., St. Louis, MO). Glass micropipettes were filled with a 10^−7^ M solution of A23187 and inserted into the arteriolar lumen, and A23187 was then infused directly into the flow stream for 2-min periods at ejection pressures of 5, 10, 20, and 40 psi (Nurkiewicz et al. 2004). A 2-min recovery period followed each ejection. At the end of all intravital experiments, adenosine (ADO) was added to the superfusate (10^−4^ M final concentration) to fully dilate the microvascular network and determine the passive diameter of each arteriole studied.

#### Protocol 2.

To evaluate arteriolar responsiveness to adrenergic stimulation, phenylephrine (PHE) was iontophoretically applied to individual arterioles in rats after exposure to either saline or 0.25 mg ROFA. Micropipettes were filled with a 50-mM solution of PHE in distilled water. The pipette tip was placed in light contact with the arteriolar wall, and a current programmer delivered continuous 2-min ejection currents of 50, 100, and 200 nA (randomly). A 2-min recovery period followed each application. To exclude the possibility that adrenergic stimulation could increase NO production and therefore attenuate the observed constrictions, these experiments were performed during NO synthase (NOS) inhibition with N^G^-monomethyl-l-arginine (10^−4^ M final superfusate concentration).

#### Protocol 3.

Adhering or rolling leukocytes in first-order venules of rats after exposure to either saline, 0.25 mg ROFA, or 0.1 mg TiO_2_ were quantified to characterize microvascular inflammation. Leukocytes that were either stationary or moving but in constant contact with the venular wall for at least 200 μm were counted for 1 min in each venule studied.

#### Protocol 4.

Oxidant activity in the arteriolar wall was measured with the tetranitroblue tetrazolium (TNBT) reduction method, which provides a general index of microvascular oxidant stress (Lenda and Boegehold 2002). After 1 hr of continuous exposure to 2% TNBT superfusion, the spinotrapezius muscle was fixed with a 10% formalin solution and excised. The tissue was then viewed with bright-field microscopy, and images of microvessels were digitized and analyzed. Using a 1 × 5 μm photometric window, a series of average pixel intensity measurements were made along the vessel wall and in extravascular regions immediately adjacent to the wall. To assess microvascular wall levels of formazan (the reduction product of TNBT and therefore an index of oxidant activity), the measured pixel intensities were used to calculate microvascular wall light absorption (*A*): *A* = ln(*l_t_*/*l_o_*), where *l_t_* is the vessel intensity and *l_o_* is the intensity for the adjacent extravascular region. The amount of formazan formed is proportional to the level of oxidant activity, and calculated light absorption is linearly related to the amount of formazan present (Lenda and Boegehold 2002).

### Data and statistical analyses.

Arteriolar diameter (*D*, in micrometers) was sampled at 10-sec intervals. Resting vascular tone was calculated for each vessel as follows: tone = [(*D*_pass_ − *D_c_*)/*D*_pass_] × 100, where *D*_pass_ is passive diameter under ADO, and *D_c_* is the diameter measured during the control period. A tone of 100% represents complete vessel closure, whereas 0% represents the passive state. All data are reported as mean ± SE, where *n* represents the number of arterioles and *N* represents the number of rats. Statistical analysis was performed by commercially available software (Sigmastat; Jandel Scientific, Chicago, IL). We used one-way repeated-measures analysis of variance (ANOVA) to determine the effect of a treatment within a group or differences among groups. Two-way repeated-measures ANOVA was used to determine the effects of group, treatment, and group × treatment interactions on measured variables. For all ANOVA procedures, we used the Student-Newman-Keuls method for post hoc analysis to isolate pairwise differences among specific groups. Significance was assessed at the 95% confidence level (*p* < 0.05) for all tests.

## Results

The general characteristics of rats used for intravital microscopy experiments are reported in Table 1. At the time of study, age and mean arterial pressure were not different among the experimental groups. Body weight was significantly higher in the 0.1-mg TiO_2_ group. Rats used for BAL data were of the same age as those reported in Table 1 (data not shown).

The effects of pulmonary exposure to ROFA and TiO_2_ on BAL parameters of inflammation and damage 24 hr after IT treatment are reported in Table 2. PMNL counts were significantly higher in the 0.1-mg TiO_2_ and 0.25-mg ROFA groups than in the saline-treated group, but not in the 0.25-mg TiO_2_ and 0.1-mg ROFA groups. BAL fluid albumin and LDH were not significantly different among the experimental groups. Total zymosan-stimulated AM CL was significantly greater in the 0.1-mg ROFA and 0.25-mg ROFA groups than that in the saline controls, whereas the 0.1-mg and 0.25-mg TiO_2_ groups were not different from the saline controls. AM counts were not statistically different among the experimental groups (data not shown).

**Figure 1:**
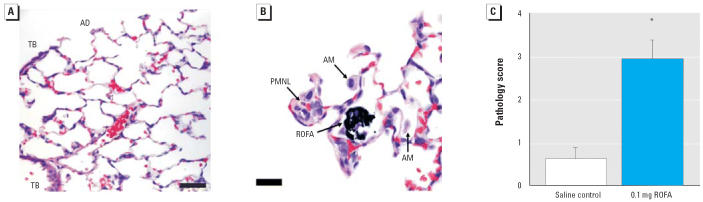
Histologic evidence of focal pulmonary alveolitis 24 hr after PM exposure. (*A*) and (*B*) are representative findings from five saline-treated rats and five rats exposed to 0.1 mg ROFA. (*A*) Saline control showing no morphologic alterations. Abbreviations: AD, alveolar duct; TB, terminal bronchioles. Bar = 50 μm. (*B*) Histopathologic alterations in a ROFA-exposed rat. Agglomerated ROFA particles in an alveolar space can be seen near an alveolar duct. The ROFA particles do not transmit light and therefore appear black when viewed in the light microscope. AMs are frequently observed in alveoli near ROFA particles and are intimately associated with the agglomerated ROFA. PMNLs are present in lesser numbers near ROFA particles, most frequently in the interstitium. Bar = 20 μm. (*C*) Mean alveolitis pathology scores. *p < 0.05 compared with saline; similar results were obtained with TiO_2_.

The pulmonary microscopic sections of five saline-treated rats and five rats exposed to 0.1 mg ROFA were examined by a board-certified veterinary pathologist at 24 hr post-exposure. Figure 1A represents a typical slide from saline-treated rats, in which no morphologic alterations are present. Erythrocytes were occasionally present in slides from saline-treated rats, but this was an artifact of the fixation technique. The alveolitis was predominantly histiocytic, although lesser numbers of neutrophils and/or eosinophils were sometimes observed. Alveolitis was generally centered around alveolar ducts or perivascular spaces near alveolar ducts in all rats. In several foci of alveolitis, agglomerated ROFA could be seen in association with alveolar inflammation (Figure 1B). The mean alveolitis pathology score of 2.92 ± 0.43 in ROFA-exposed rats was significantly greater than that for saline-treated rats (0.60 ± 0.27; Figure 1C).

Resting variables of all arterioles studied 24 hr postexposure are reported in Table 3. Resting and passive (in the presence of ADO) arteriolar diameters were not significantly different among the experimental groups. Accordingly, resting arteriolar tone was not different among the experimental groups.

**Figure 2:**
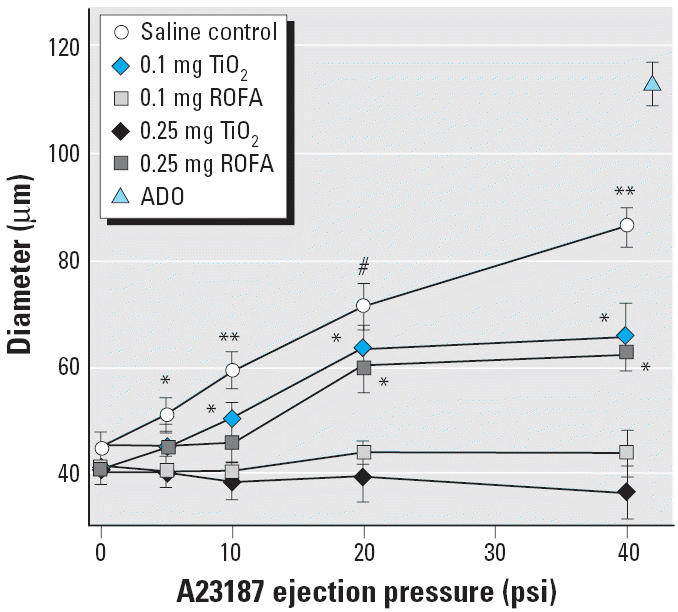
PM exposure impairs or abolishes spinotrapezius muscle arteriolar responsiveness to intraluminal A23187 in a dose-dependent manner 24 hr after IT treatment. *n*, number of arterioles. Saline control, *n* = 13; 0.1 mg TiO_2_, *n* = 9; 0.1 mg ROFA, *n* = 9; 0.25 mg TiO_2_, *n* = 8; 0.25 mg ROFA, *n* = 8. Maximum diameter obtained with ADO (10^−4^ M, final superfusate concentration). Values are mean ± SE. *p < 0.05 compared with 0.25 mg TiO_2_ and 0.25 mg ROFA. **p < 0.05 compared with 0.1 mg TiO_2_, 0.1 mg ROFA, 0.25 mg TiO_2_, and 0.25 mg ROFA. #*p* < 0.05 compared with 0.1 mg ROFA, 0.25 mg TiO_2_, and 0.25 mg ROFA.

In spinotrapezius muscle arterioles of the saline-treated group, A23187 infusion produced dose-dependent dilation that was near maximal at the highest ejection pressure (Figure 2). Exposure to 0.25 mg TiO_2_ or ROFA completely abolished this response 24 hr postexposure at each ejection pressure. Arterioles in rats exposed to 0.1 mg TiO_2_ or ROFA displayed an attenuated responsiveness to A23187 infusion. In these groups, vaso-dilation in response to A23187 infusion at 20 and 40 psi was significantly greater than that observed in rats exposed to either particle at 0.25 mg. Additionally, the response in the 0.1 mg TiO_2_ group at 10 psi was significantly greater than that observed for either group at 0.25 mg. These findings support our original observations and are consistent with our postulate that pulmonary exposure to PM inhibits systemic microvascular function in a dose-dependent manner (Nurkiewicz et al. 2004).

**Figure 3:**
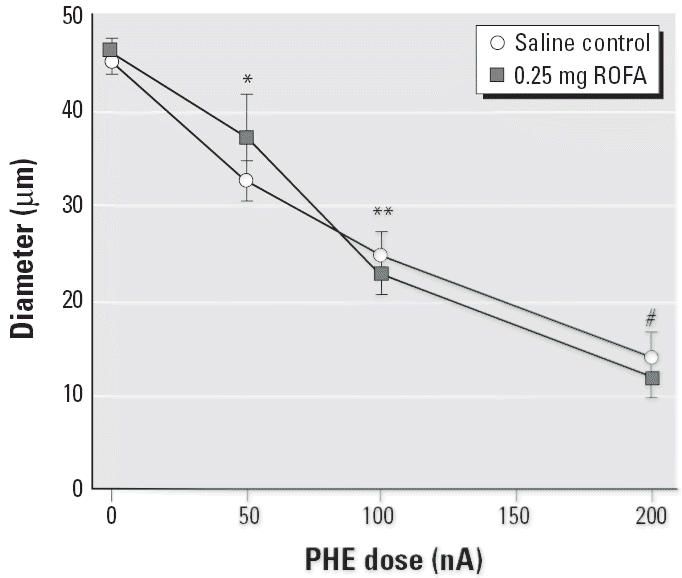
ROFA exposure does not alter arteriolar vasopressor responsiveness to PHE in the spinotrapezius muscle. *n*, number of arterioles. Saline control, *n* = 8; 0.25 mg ROFA, *n* = 8. Values are mean ± SE. *p < 0.05 compared with 0 nA in both groups; **p < 0.05 compared with 50 nA in both groups; #*p* < 0.05 compared with 100 nA in both groups.

Arteriolar adrenergic sensitivity 24 hr after PM exposure was assessed with PHE application and resultant vasoconstriction (Figure 3). Iontophoretic PHE application produced robust, dose-dependent arteriolar constriction in saline-treated rats. In rats exposed to 0.25 mg ROFA (a pulmonary load that abolished endothelium-dependent dilation), the arteriolar responses to PHE iontophoresis were identical to those in saline-treated rats. This suggests that after pulmonary PM exposure, peripheral arterioles are not hypersensitive to adrenergic stimulation and that their contractile ability is unaltered.

**Figure 4:**
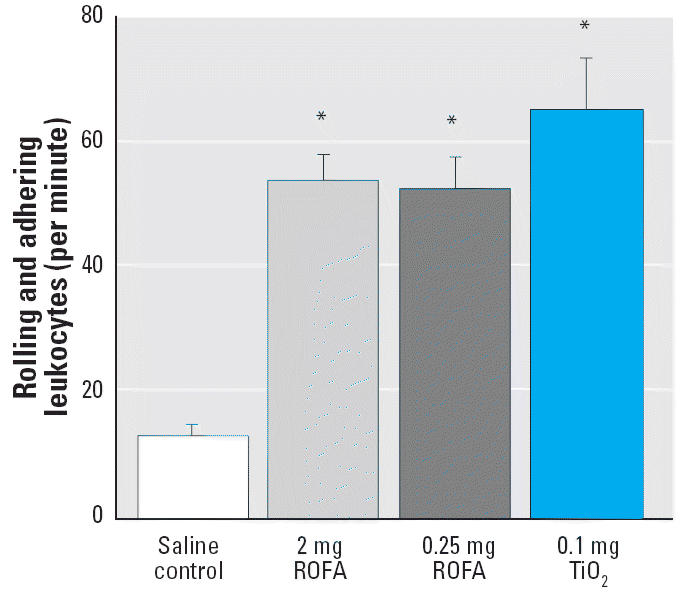
PM exposure increases venular leukocyte rolling and adhesion in the spinotrapezius muscle 24 hr after IT treatment. Venular leukocytes were quantified as rolling and adhering leukocytes per minute in a 200-μm segment. *n*, number of venules. Saline control, *n* = 26; 2 mg ROFA, *n* = 15; 0.25 mg ROFA, *n* = 18; 0.1 mg TiO_2_, *n* = 10. Values are mean ± SE. *p < 0.05 compared with saline control.

The number of rolling and adherent leukocytes through 200 μm venular segments is displayed in Figure 4. We have previously reported this number to be as great as 54 ± 4 leukocytes/min in rats exposed to 2 mg ROFA (Nurkiewicz et al. 2004). This systemic response to PM exposure is widespread throughout the microvascular network and is significantly greater than that observed in venules of saline-treated rats (13 ± 2 leukocytes/min). Results in the present study indicate that, at 24 hr postexposure, this dynamic response persists at equivalent magnitudes in venules of rats exposed to either 0.25 mg ROFA or 0.1 mg TiO_2_ (52 ± 5 or 65 ± 9 leukocytes/min, respectively).

**Figure 5:**
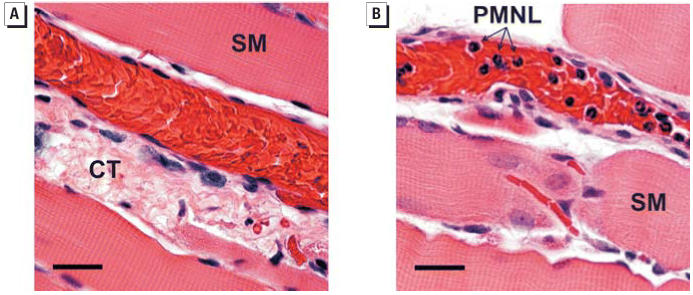
PMNL identification in the spinotrapezius muscle microcirculation of PM-exposed rats 24 hr after IT exposure. (*A*) Representative H&E-stained section from a saline-treated rat. Abbreviations: CT, connective tissue; SM, skeletal muscle fiber. (*B*) Representative H&E-stained section from a rat exposed to 0.1 mg ROFA. Note the deeply lobed nuclei that are characteristic of PMNLs. Bars = 25 μm; similar results were obtained with TiO_2_.

Spinotrapezius muscle histology was performed to specifically identify the adherent and rolling leukocytes reported in Figure 4. Rats were either treated with saline or exposed to 0.1 mg ROFA. At 24 hr postexposure, the muscles were excised, fixed, and stained with H&E (Figure 5). Histologic analysis positively identified the adherent cells in venules from ROFA-exposed rats as PMNLs because of the presence of deeply lobed nuclei. Identical results were obtained in rats exposed to TiO_2_ (data not shown).

**Figure 6:**
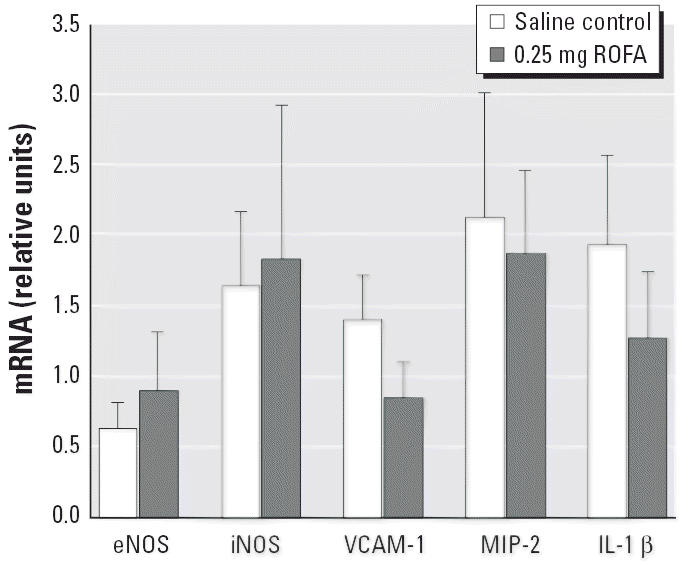
Microvascular inflammatory markers 24 hr after exposure to ROFA: mRNA measurements in microvessels from six saline-treated rats and six rats exposed to 0.25 mg ROFA. After microdissection, vessels from both spinotrapezius muscles of an individual rat were pooled for data collection. Abbreviations: MIP-2, macrophage inflammatory protein; VCAM-1, vascular cell adhesion molecule. Note that neither eNOS nor iNOS message was altered after ROFA exposure.

RT-PCR was performed to characterize potential inflammatory markers at the systemic microvascular level after PM exposure (Figure 6). Rats were either treated with saline or exposed to 0.25 mg ROFA 24 hr before spinotrapezius muscle microvessel dissection. Although no inflammatory marker was different between the two groups, it is important to note that neither endothelial NOS (eNOS) nor inducible NOS (iNOS) message was altered by PM exposure. This suggests that the capacity of microvascular endothelium to synthesize eNOS is not impaired after PM exposure.

**Figure 7:**
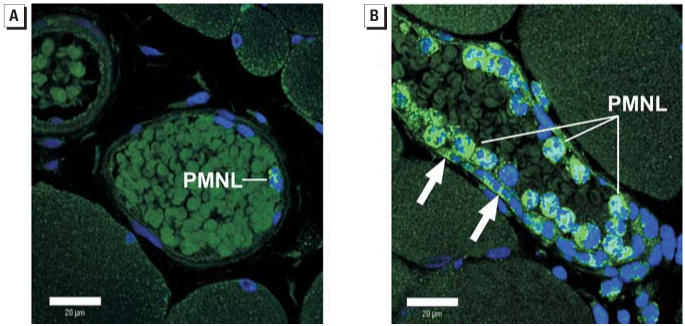
Localization of MPO in the spinotrapezius muscle microcirculation 24 hr after ROFA exposure. Fluorescent antibodies targeted a polyclonal antibody against MPO; nuclei are counterstained blue with DAPI. (*A*) Representative confocal fluorescent image of a venule from a saline-treated rat. (*B*) Representative confocal image of a venule from a rat exposed to 0.25 mg ROFA. Note the fluorescence in the microvascular wall indicating the presence of MPO (arrows). Bars = 20 μm; similar results were obtained with TiO_2_.

To further characterize inflammatory events associated with venular PMNL adhesion 24 hr after PM exposure, we identified the presence of MPO in neutrophils and the microvascular wall in the spinotrapezius muscle (Figure 7). Representative confocal fluorescence images are presented in Figure 7. In Figure 7A, MPO is evident (intense green fluorescence) in a single PMNL of a saline-treated rat. In Figure 7B, MPO is evident not only in each of the multiple PMNLs but also in the microvascular wall of a rat exposed to 0.25 mg ROFA. It is also apparent in Figure 7B that some PMNLs have migrated, or are in the process of migrating, from the microvascular lumen into the interstitial space. Similar results were observed in rats exposed to TiO_2_ (data not shown). This histologic and immunologic evidence suggests that MPO deposition occurs in the systemic microvascular wall after pulmonary PM exposure.

**Figure 8:**
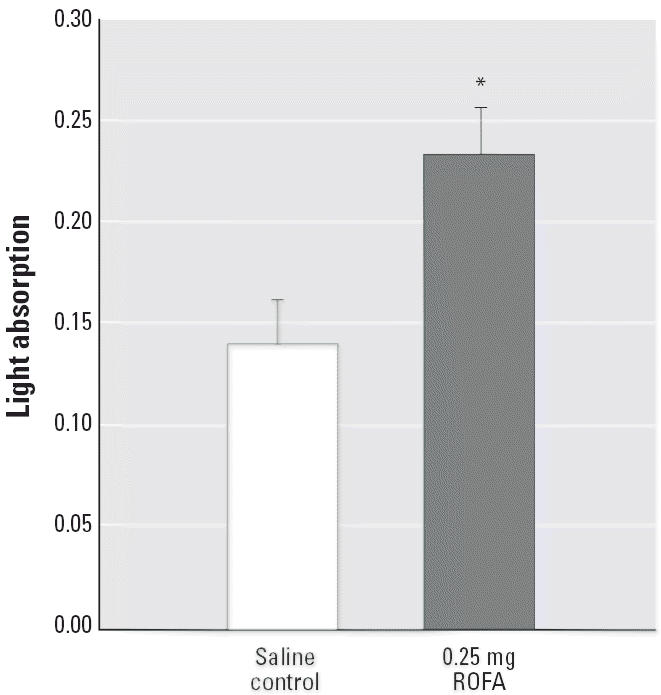
ROFA exposure increases oxidative stress in the systemic microcirculation 24 hr after IT treatment. *n* = number of vessels. Calculated wall light absorption [*A* = ln(*l_t_*/*l_o_*)] in microvessels from saline-treated rats (*n* = 16) and rats exposed to 0.25 mg ROFA (*n* = 15) after exposure to 2% TNBT. Values are mean ± SE. *p < 0.05 compared with saline control.

To better characterize the effects of systemic inflammation associated with PM exposure, general oxidative stress was measured in the spinotrapezius muscle microvascular wall 24 hr after IT treatment (Figure 8). Calculated light absorption (from deposits of formazan, the reduction product of TNBT and reactive oxygen species) in the microvascular wall from rats exposed to 0.25 mg ROFA was significantly greater than that from saline-treated rats. This suggests that general oxidative stress in the systemic microcirculation increases after pulmonary PM exposure.

## Discussion

This second report from our group is part of our ongoing investigation of the remote biologic effects at the systemic microvascular level that follow pulmonary PM exposure. In the present study, we present three novel observations. Additionally, we have verified our previous findings using lower PM doses.

We have previously reported that exposure to ROFA produces a dose-dependent impairment of systemic endothelium-dependent arteriolar dilation and increases venular leukocyte adhesion and rolling (Nurkiewicz et al. 2004). This arteriolar impairment is equally present after exposure to identical doses of TiO_2_ (Figure 2). Similarly, leukocyte adhesion and rolling remain elevated at lower doses of ROFA and TiO_2_ (Figure 4). These findings reinforce our postulate that the remote biologic effects at the systemic microvascular level after PM exposure are due to the presence of particles in the lung, rather than their inherent pulmonary toxicity, because BAL markers of lung damage were not elevated at doses of < 0.25 mg PM/rat (Table 2, albumin and LDH).

Consistent with our previous study (Nurkiewicz et al. 2004), we report here that systemic microvascular responses after PM exposure are independent of the degree of pulmonary inflammation (as determined by BAL). This is evident from the data in Table 2: BAL from rats exposed to either ROFA or TiO_2_ or treated with saline is neither predictably different nor wholly convincing in areas where significance is noted (Table 2, PMNL). However, because activated inflammatory cells may adhere to adjacent tissues or form aggregates too large to recover by BAL or simply involve a very small fraction of the lung, isolated pulmonary “hot spots” may not be represented by our BAL data. Therefore, lung tissue was examined for histopathologic changes by a pathologist to better identify pulmonary pathology after PM exposure. The data in Figure 1 indicate that such “hot spots,” or foci of histiocytic alveolitis, are associated with deposition sites of PM. The collective impression was that this inflammation was focal rather than diffuse (Figure 1C). The increased macrophage activation associated with these foci may be indicated by the dose-responsive increase in AM CL, a measure of macrophage activation.

Several reports suggest that the autonomic influence on a given tissue is altered after PM exposure (Brook et al. 2004; Chen and Hwang 2005; Wichers et al. 2004). In the present study, we did not observe any differences in blood pressure (Table 1) or arteriolar tone (Table 3). Although this observation does not support the postulate that autonomic activity is altered after PM exposure, it is limited because our experimental data were collected in anesthetized rats. Given this limitation, neurogenic input may still be enhanced by increased receptor sensitivity. The first major finding we report here is that systemic arteriolar α_1_-adrenergic receptor sensitivity is unaltered after PM exposure. This is evident in Figure 3, which shows identical arteriolar constriction produced by iontophoretic application of PHE in both saline-treated and ROFA-exposed rats.

Translocation of PM from the lung to remote sites may also be responsible for adverse cardiovascular effects (Calderon-Garciduenas et al. 2001; Nemmar et al. 2004; Oberdorster et al. 2004). In the present study, ROFA and TiO_2_ particles of at least 1 μm were used, and systemic microvascular changes were measured 24 hr postexposure. There is currently no evidence suggesting that fine PM migrates to systemic sites within this time frame. However, microvascular TiO_2_ deposition will be addressed in future studies. A second translocation possibility is that soluble metals from ROFA reach the systemic microcirculation. Soluble metals have been shown to drive many of the pulmonary effects of ROFA (Dreher et al. 1997; Kodavanti et al. 1998; Rice et al. 2001). However, because TiO_2_ exhibits the same dose dependence as ROFA (Figures 2 and 4), soluble metals do not appear to be driving the reported microvascular effects.

Our original observations of increased venular leukocyte adhesion and rolling were made in rats exposed to 2 mg ROFA (Nurkiewicz et al. 2004). This effect is repeatable after exposure to either 0.25 mg ROFA or 0.1 mg TiO_2_ (Figure 4). Because this robust response is independent of the PM type or dose, other types of PM may elicit a less pronounced response at lower doses than those used here.

Intravital microscopy is a powerful tool that allows direct observation of leukocyte–venule interaction *in vivo*, but the maximum optical resolution of our system is approximately 1 μm. Although leukocytes can be easily identified by their characteristic rolling and adhesion in a laminar stream of red cells, further characterization is not possible at this resolution. This issue was resolved via histology of the spinotrapezius muscle (Figure 5), which allowed us to identify the adhering and rolling leukocytes as PMNLs.

Our PCR results indicate that the messages for adhesion factors at the microvascular level are not altered after PM exposure (Figure 6). This suggests that such factors are not altered after exposure to PM, but it is possible that the message quickly increases and then subsides within the 24 hr postexposure period we used. It is also possible that other adhesion factors are involved and/or that the adhesion factors on the leukocytes are altered with no change in the endothelial expression of such factors. Our PCR results also indicated that microvascular eNOS and iNOS messages are not altered after PM exposure. A decrease in eNOS message could have been the cause of the impaired endothelium-dependent dilation, and an increase in iNOS message could have been responsible for the observed microvascular inflammation. However, data shown in Figure 6 do not support the hypothesis that eNOS or iNOS is altered after PM exposure.

The second major finding in this study is that microvascular MPO deposition is associated with PM exposure (Figure 7). Lipopolysaccharide injection in rats produces a diffuse localization of MPO throughout the aortic endothelium (Eiserich et al. 2002). The MPO was presumed to be secreted by activated leukocytes and taken up by endothelial cells and vascular tissue, independent of neutrophil extravasation. We identified MPO in neutrophils and in the microvasculature of the rat spinotrapezius muscle after treatment of rats with ROFA or TiO_2_. Although the MPO was not found in all vessels, it was localized primarily in microvessels within the vicinity of adherent or migrating neutrophils. Given the anatomical and physiologic differences between the aorta and the microcirculation, as well as the heterogeneous nature of a microvascular network, differences in MPO localization are not unexpected. In the spinotrapezius microvasculature, deposition of MPO may also occur during transmigration of the neutrophils. Further immunohistochemical experiments are being done to distinguish endothelial cells from neutrophils because it is possible that the staining we see is not in the endothelial cells but in transmigrating neutrophils. It is also important to note that MPO was identified at a single time point, and future studies must characterize the temporal relationship between PM exposure and MPO deposition. Moreover, future studies will determine the contribution of local MPO deposition to microvascular dysfunction associated with PM exposure.

MPO is the most abundant hemoprotein in leukocytes and comprises approximately 5% of their dry weight (Klebanoff 2005). Therefore, identification of MPO in PMNLs of saline-treated or ROFA-exposed rats is not novel. However, given the significant increase in rolling and adherent PMNLs after ROFA exposure (Figures 4 and 5), it is likely that the systemic microvascular inflammation may be triggered by MPO. This is further supported by the observation that firm adhesion of leukocytes to the venular wall is not necessary to alter endothelial intracellular Ca^2+^ concentration or microvascular permeability (Zhu et al. 2005). Moreover, MPO was identified in the microvascular wall of ROFA-exposed rats, whereas it was absent in that of saline-treated rats (Figure 7). Upon deposition in the vascular wall, MPO is preferentially situated in the subendothelial matrix (Baldus et al. 2001), a position in which MPO can ideally interrupt NO signaling between the endothelium and the vascular smooth muscle. MPO may disrupt the influence of NO on microvascular tone from at least two perspectives. MPO generates reactive substrate radicals that consume NO (Eiserich et al. 2002). Alternatively, MPO produces hypochlorous acid that can chlorinate l-arginine and render it unusable by NOS as a substrate for NO production (Zhang et al. 2001).

The third major finding in this study is that oxidative stress is increased in the microvascular wall after PM exposure (Figure 8). The TNBT assay is useful for characterizing general oxidative stress, but it cannot identify specific reactive oxygen species. However, given that leukocyte adhesion and rolling are markedly increased after ROFA exposure (Figure 5) and that MPO is present in the cells and microvascular wall (Figure 7), it is likely that hydrogen peroxide and superoxide are involved in this process. Regardless of which oxygen radicals are elevated after PM exposure, such radicals in the vascular wall have been shown to alter vascular tone and impair endothelium-dependent arteriolar dilation (Lenda et al. 2000; Sato et al. 2003).

Alterations in the vascular reactivity and resting diameter of a large conduit artery after PM exposure have been reported (Brook et al. 2002; O’Neill et al. 2005). Studies by O’Neill et al. (2005) investigated human brachial artery reactivity in diabetic humans after PM exposure and suggested that both endothelium-dependent and -independent arterial dilation are impaired after PM exposure. Conversely, studies by Brook et al. (2002) have reported no change in these vascular reactivity indexes, but they did suggest that the brachial artery constricts by 0.09 ± 0.15 mm after PM exposure. This subtle change had no effect on peripheral resistance because neither systolic nor diastolic blood pressure was altered. O’Neill et al. (2005) reported that endothelium-dependent dilation produced an approximately 6% increase in brachial artery diameter under normal conditions, and this response is decreased by approximately 9% after PM exposure. If we assume a resting brachial artery diameter of 4 mm (Brook et al. 2002), endothelium-dependent dilation under the conditions reported by O’Neill et al. (2005) would cause the brachial artery to dilate to approximately 4.24 mm, and PM exposure would attenuate this dilation to approximately 4.22 mm. The relative contribution of these nominal PM-dependent effects on peripheral resistance would be negligible in a vascular segment that provides little to no vascular resistance. Furthermore, neither study reported brachial artery blood flow data after PM exposure, which would provide some insight into downstream changes in the resistance vasculature. Although the findings by O’Neill et al. (2005) and Brook et al. (2002) do provide valuable biologic end points, the absence of a link to the resistance vasculature makes the physiologic relevance of these findings difficult to assess.

If changes in the reactivity of conduit arteries are not responsible for PM-associated cardiovascular morbidity and mortality, then what vascular events could precipitate such an outcome? Cardiac disturbances and decreases in arterial pressure may occur after pulmonary PM exposure (Wichers et al. 2004). These changes do not appear to be outwardly consistent with our model, in which PM exposure compromises the capacity of the systemic arterioles to dilate. One explanation for this paradox may be that an acute baroreceptor reflex is occurring in response to an increased cardiac afterload. During periods of activity (e.g., walking or stair climbing), cardiac output increases and peripheral dilation is essential to match the metabolic needs of active tissues. An inability to sufficiently reduce peripheral resistance in these circumstances would augment cardiac afterload, thus further increasing arterial pressure. This increased arterial pressure could stimulate an acute baroreceptor reflex that would decrease cardiac output and therefore arterial pressure.

Increases in arterial pressure after PM exposure have also been documented (Chang et al. 2004; Vincent et al. 2001). Our data do not indicate that PM exposure increases arterial pressure, but this possibility cannot be discounted because our data were collected from anesthetized rats. Regardless, the relationship between our findings and those that indicate arterial pressure is increased after PM exposure is more evident. In this case, if our findings in the spinotrapezius muscle are representative of PM exposure effects in other microvascular beds, the inability to decrease peripheral resistance would directly contribute to an increased arterial pressure. In some susceptible populations, such as those with hypertension or vascular disease, this increase in arterial pressure could be fatal if appropriate compensatory mechanisms are also compromised.

## Conclusions

The present findings verify our previous report (Nurkiewicz et al. 2004) that systemic endothelium-dependent arteriolar dilation is impaired after pulmonary PM exposure in a dose-dependent manner. Local MPO deposition and oxidative stress may be mechanisms by which this effect occurs. These findings are consistent with the larger body of evidence that suggests systemic inflammation follows pulmonary PM exposure. Future mechanistic studies will identify the relative contribution of these two effects to the established microvascular dysfunction. It will also be important to determine if these inflammatory effects are localized to target tissues or part of a larger systemic response. The data presented here also suggest that arteriolar adrenergic sensitivity is not affected by PM exposure. Although the systemic microcirculation is capable of overriding neurogenic input, such input still plays a major role in the collective generation of vascular resistance and blood flow distribution. Further studies are necessary to better clarify the influence of peripheral nerves on microvascular function after PM exposure and to determine if this is consistent with a baroreceptor reflex. Because autonomic reflexes can occur rapidly, it will be essential to characterize the temporal relationship of such reflexes after PM exposure.

## Figures and Tables

**Table 1:** Profiles of experimental animals used for intravital studies.

Experimental group	*N*	Age (days)	Weight (g)	Mean arterial pressure (mm Hg)
Saline control	15	55 ± 4	236 ± 4	98 ± 5
0.1 mg TiO_2_	5	50 ± 1	275 ± 9*	108 ± 6
0.1 mg ROFA	4	52 ± 1	225 ± 3	102 ± 9
0.25 mg TiO_2_	3	56 ± 3	219 ± 7	91 ± 15
0.25 mg ROFA	11	50 ± 2	221 ± 8	94 ± 7

*N*, number of rats. Values are mean ± SE.

* *p* < 0.05 compared with all other groups.

**Table 2:** BAL data from saline-treated and TiO_2_- and ROFA-exposed rats.

		BAL fluid		
Experimental group	Cellular content of PMNL (10^6^ cells/rat)	Albumin (mg/mL)	LDH (U/L)	Total AM CL
Saline control	0.93 ± 0.11	0.13 ± 0.02	58 ± 10	7.50 ± 1.63
0.1 mg TiO_2_	1.73 ± 0.36*	0.14 ± 0.04	67 ± 13	5.21 ± 1.60
0.1 mg ROFA	1.24 ± 0.28	0.23 ± 0.05	75 ± 2	14.47 ± 2.60*^,^**
0.25 mg TiO_2_	1.11 ± 0.15	0.19 ± 0.02	65 ± 8	3.64 ± 0.73
0.25 mg ROFA	1.91 ± 0.20*	0.17 ± 0.01	46 ± 4	17.42 ± 1.11*^,^**

*N* = 22 rats for saline control; *N* = 5–7 rats for all other doses. Values are mean ± SE. CL = counts per minute × 10^5^/0.25 × 10^6^ AM/15 min.

* *p* < 0.05 compared with saline.

** *p* < 0.05 compared with 0.1 mg TiO_2_ and *p* < 0.05 compared with 0.25 mg TiO_2_.

**Table 3:** Resting variables for all arterioles studied (mean ± SE).

	Saline	0.1 mg TiO_2_	0.1 mg ROFA	0.25 mg TiO_2_	0.25 mg ROFA
No. of arterioles	28	9	9	8	25
Resting diameter (μm)	44 ± 2	45 ± 1	41 ± 2	41 ± 2	43 ± 1
Passive diameter (μm)	108 ± 3	111 ± 4	111 ± 6	100 ± 3	106 ± 3
Resting tone (% of maximum)	59 ± 2	59 ± 2	62 ± 3	59 ± 2	59 ± 1
